# Research Output and Global Trends in Lassa Fever: A 50‐Year Bibliometric Analysis (1975–2025)

**DOI:** 10.1155/bmri/6790037

**Published:** 2026-08-12

**Authors:** Ifeanyi Ferdinand Chukwuma, Chioma Miracle Ojiako, Simon Pierre Yinyang Danga, Kenechukwu David Oli, Ruth Asikiya Afunwa, Angus Nnamdi Oli

**Affiliations:** ^1^ Department of Pharmaceutical Microbiology and Biotechnology, Faculty of Pharmaceutical Sciences, Nnamdi Azikiwe University, Awka, Anambra State, Nigeria, unizik.edu.ng; ^2^ Department of Pharmaceutical Microbiology and Biotechnology, Faculty of Pharmaceutical Sciences, Federal University Oye Ekiti, Oye Ekiti, Ekiti State, Nigeria, fuoye.edu.ng; ^3^ School of Science, Technology and Engineering, University of the Sunshine Coast, Sippy Downs, Queensland, Australia, usc.edu.au; ^4^ Department of Biological Sciences, Faculty of Science, University of Garoua, Garoua, Cameroon; ^5^ Federal Medical Center, Makurdi, Benue State, Nigeria; ^6^ Department of Pharmaceutical Microbiology and Biotechnology, Faculty of Pharmaceutical Sciences, Chukwuemeka Odumegwu Ojukwu University, Igbariam, Anambra State, Nigeria, coou.edu.ng

**Keywords:** bibliometric analysis, global trends, Lassa fever, research output

## Abstract

**Background:**

Lassa fever poses a significant public health threat, primarily concentrated in West Africa. This bibliometric study aimed to document research outputs concerning Lassa fever. It analyzes trends in research publication output, the participation of various countries, productive institutions and their collaboration networks, influential funding agencies, predominant research fields, top‐tier journals, prolific authors, and keyword frequencies.

**Methods:**

Lassa fever‐related publications published between 1975 and 2025 were retrieved from the Scopus and Web of Science (WoS) databases by using the keywords “Lassa fever,” OR “Lassa virus,” OR “Lassa hemorrhagic fever,” OR “Lassa mammarenavirus,” OR “LASV.” The retrieved records were restricted to the study period, screened for English language, and further refined to include original research articles. The Scopus and WoS datasets were analyzed independently using Biblioshiny (Bibliometrix package in RStudio Cloud) to evaluate research productivity and trends, including contributions by authors, countries, institutions, and journals, whereas VOSviewer (Van Eck & Waltman, Leiden University, Netherlands) was used for network visualization, construct collaboration, and keyword co‐occurrence networks. Country productivity was assessed using the full‐counting approach based on authors′ institutional affiliations.

**Results:**

Following the application of the eligibility criteria, 1776 and 2451 English‐language original research articles on Lassa fever from Scopus and WoS databases, respectively, were included in the bibliometric analysis. The findings demonstrated an overall increase in research output during the study period (1975–2025), with publication output peaking in 2024. The United States and Nigeria were the leading contributors to Lassa fever research in both databases. Despite the endemicity of Lassa fever in West Africa, only a few number of other African countries were represented among the top 20 global research contributors. Bernhard‐Nocht‐Institut für Tropenmedizin and the Centers for Disease Control and Prevention were the most prolific institutions in both databases. The National Institute of Allergy and Infectious Diseases (Scopus) and the US Department of Health and Human Services (WoS) emerged as the leading funding agencies. “Medicine” (Scopus) and “Infectious Diseases” (WoS) were the predominant research fields. The most prolific authors included Günther S., De La Torre J.C., and Garry R.F., whereas the *Journal of Virology* was the leading publication source in both databases. Keyword analysis identified “Lassa fever” and “Lassa virus” as the most frequent occurring terms.

**Conclusion:**

The bibliometric analysis provides an updated, comprehensive historical perspective of progress in Lassa fever research. It also highlighted the pivotal role of diverse stakeholders. However, African research output is disproportionately low in relation to that of other countries, implying a need for increased funding and focus on Lassa fever research for effective prevention and control.

## 1. Background

Bibliometric analysis is a quantitative research tool that systematically employs statistical and mathematical techniques to evaluate scientific publications and characterize the development of research within a specific field in order to gain comprehensive insights [1]. It serves as a valuable technique for the identification of growth trends, subject distribution, significant keywords, influential authors, leading institutions, countries, journals, collaboration networks, highly cited publications, and emerging research themes. Consequently, bibliometric analysis has become an indispensable tool for evaluating scientific productivity, identifying research hotspots, guiding funding priorities, and informing evidence‐based decision‐making in biomedical research [2, 3]. Furthermore, bibliometric and evidence‐synthesis approaches have been widely applied across infectious disease research to evaluate scientific progress, identify knowledge gaps, and guide future research priorities, including studies investigating the bacteriology and virulence factors associated with neonatal infections [4]. Widely used bibliometric software such as Bibliometrix and VOSviewer further facilitate science mapping and visualization of intellectual structures and collaborative relationships within research fields [1–3]. Similar bibliometric investigations have successfully characterized the evolution of research on major infectious diseases, including Ebola virus disease and coronavirus disease 2019 (COVID‐19), demonstrating the usefulness of this approach in understanding rapidly expanding scientific literature [5, 6].

Lassa fever is an acute viral hemorrhagic disease caused by Lassa virus, an enveloped, bisegmented, single‐stranded negative‐sense RNA virus belonging to the genus *Mammarenavirus*. First recognized in 1969 following a nosocomial outbreak in Lassa, northeastern Nigeria, the disease has since become one of the most important endemic viral hemorrhagic fevers in West Africa [7–11]. Despite more than five decades of investigation, Lassa fever remains a significant public health challenge because of its recurrent outbreaks, substantial morbidity and mortality, and persistent socioeconomic burden in endemic communities [10–12].

The disease is endemic in several West African countries, particularly Nigeria, Sierra Leone, Liberia, Guinea, and Benin, where recurrent outbreaks continue to place considerable pressure on already fragile healthcare systems [8, 10, 11]. It is estimated that between 200,000 and 500,000 infections occur annually, resulting in thousands of deaths each year, although the true burden is likely underestimated because of inadequate surveillance, limited diagnostic capacity, and underreporting in many endemic regions [10, 11]. Furthermore, increasing international travel has resulted in imported cases outside Africa, highlighting the global relevance of Lassa fever and the potential for cross‐border transmission [10].

Transmission of Lassa virus occurs through multiple pathways. The principal route is zoonotic transmission from the multimammate rat (*Mastomysnatalensis*), the natural reservoir of the virus, which maintains persistent asymptomatic infection and continuously sheds virus in urine, feces, and saliva, contaminating the environment and household food supplies [8, 13, 14]. Human‐to‐human transmission occurs primarily through direct contact with infected blood, secretions, tissues, or other bodily fluids, with nosocomial transmission representing a major source of infection among healthcare workers during outbreaks [9]. These diverse transmission pathways have contributed to the continued persistence and periodic resurgence of Lassa fever across endemic regions.

Clinically, Lassa fever presents with a broad spectrum of manifestations ranging from mild febrile illness to severe multisystem disease accompanied by hemorrhage, shock, neurological complications, and multi‐organ failure [7, 10]. Sensorineural hearing loss is among the most characteristic long‐term complications, affecting a substantial proportion of survivors regardless of disease severity [15]. Although ribavirin remains the principal antiviral therapy and has demonstrated greatest benefit when administered early during infection, treatment options remain limited, and no licensed vaccine is currently available for human use [7].

Although Lassa fever primarily affects West Africa, it is still a critical global health issue due to the possibility of cross‐border transmission [16]. Therefore, it becomes crucial to collectively quantify and evaluate research and provide an updated, comprehensive, and historical perspective on Lassa fever. The present study thus aimed to quantify and map the scientific output of research on Lassa fever published between 1975 and 2025 using a bibliometric perspective. It analyzes trends in research publication output, participation of various countries, productive institutions and their collaboration networks, influential funding agencies, predominant research fields, top‐tier journals, prolific authors, and keywords.

## 2. Materials and Methods

### 2.1. Study Design

This study employed bibliometric analysis, a technique that has increasingly been used as a tool to monitor the research performance of scientific disciplines and to support appropriate policy actions [1–3].

### 2.2. Data Source

The study used the Scopus and Web of Science (WoS) databases. The selected databases cover most of the key international journals. Since the data used in this study were retrieved from public databases and involved no direct interaction with human or animal subjects, ethical approval was not required.

### 2.3. Search Strategy

The study used the following keywords: “Lassa fever,” “Lassa virus,” “Lassa hemorrhagic fever,” “Lassa mammarenavirus,” or “LASV” to retrieve Lassa fever documents from Scopus and WoS published between 1975 and December 31, 2025. These keywords were searched in the article titles to maximize the accuracy of the retrieved research output. In order to include all published documents, the basic search method was used.

### 2.4. Inclusion and Exclusion Criteria

#### 2.4.1. Inclusion Criteria

The study included peer‐reviewed original research articles on Lassa fever published between 1975 and 2025 and indexed in the Scopus and WoS Core Collection databases. Only articles published in English were considered. Publications were required to focus primarily on Lassa fever and to contain sufficient bibliographic information (e.g., title, authors, abstract, keywords, publication year, journal, affiliations, and citations) for bibliometric analysis.

#### 2.4.2. Exclusion Criteria

The following records were excluded from the analysis: review articles, editorial materials, conference papers and proceedings, letters, notes, book chapters, books, corrections, retracted publications, meeting abstracts, early‐access records lacking complete bibliographic information, duplicate records, and publications not primarily related to Lassa fever. In addition, non‐English publications were excluded to maintain consistency in the bibliometric dataset, although this may have resulted in the omission of relevant studies from Francophone Lassa fever‐endemic countries and introduced potential language (Anglophone) bias.

### 2.5. Data Extraction

Following eligibility screening, bibliographic records were exported separately from the Scopus and WoS databases. Each database was processed separately, and records were screened for duplicate entries using Mendeley, followed by manual verification and confirmation in Zotero. No merged dataset was created, and the Scopus and WoS datasets were analyzed independently. The cleaned bibliographic records were then exported for subsequent bibliometric analysis. The extracted data included publication title, authors, year of publication, journal, author affiliations, countries/territories, abstracts, author keywords, indexed keywords, citation counts, and references. Only English‐language original research articles meeting the predefined eligibility criteria were retained for the final bibliometric analysis.

### 2.6. Bibliometric Analysis

This study mainly reported descriptive statistics. The research trends and selected publication features from both databases were separately classified and analyzed using the “Biblioshiny” app (RStudio Cloud) [17], after which they were compared. These included the distribution of the most productive countries/territories, institutions, authors, research fields, journals, and keywords. Country productivity was assessed using the full counting approach based on authors′ institutional affiliations; therefore, publications involving authors from multiple countries were counted once for each participating country, resulting in cumulative country counts exceeding the total number of publications. In this regard, the journal impact factors and total citations were obtained from the “Journal Citation Reports (JCR) Ranking: 2019” [18]. VOSviewer (Van Eck & Waltman, Leiden University, Netherlands) was used for network visualization, construct collaboration, and keyword co‐occurrence networks.

## 3. Results

**Figure 1:**
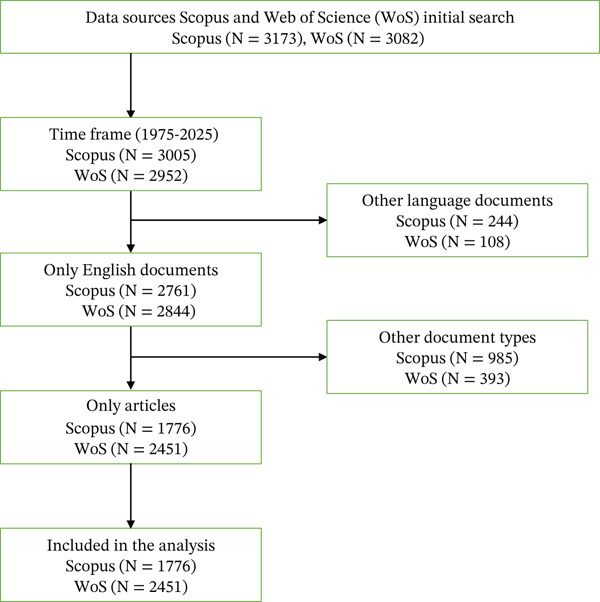
Flowchart of the methodology.

Using the search strategy in both Scopus and WoS, TITLE: (Lassa fever) OR TITLE: (Lassa virus) OR TITLE: (Lassa hemorrhagic fever) OR TITLE: (LASV) OR TITLE: (Lassa mammarenavirus), we identified 2761 and 2844 English‐language documents from Scopus and WoS, respectively, published between 1975 and 2025. The retrieved English‐language documents were subsequently screened according to the study eligibility criteria, and only original research articles were retained for the bibliometric analysis. Consequently, a total of 1776 Scopus records and 2451 WoS records were included in the final analysis (Figure 1).

### 3.1. Annual Trends in Publications

**Figure 2:**
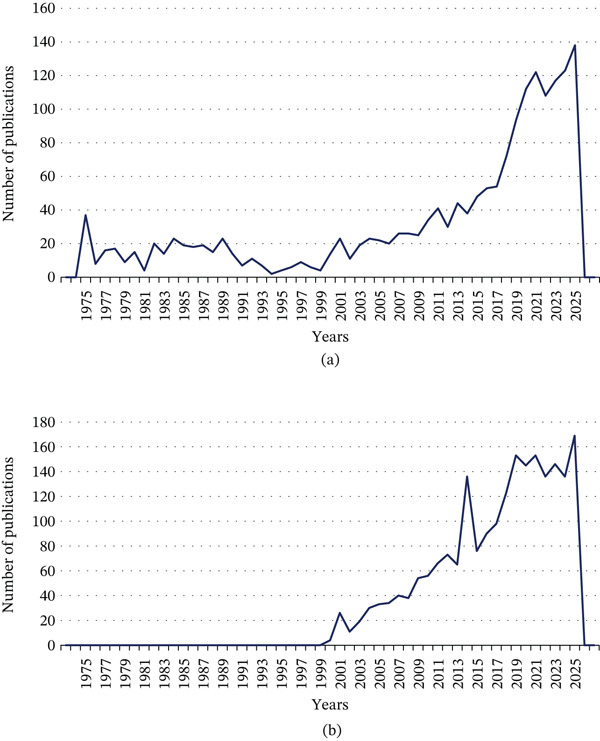
Annual trend of research publications as indexed in (a) Scopus and (b) WoS (1975–2025).

The annual publication trend observed in both Scopus and WoS demonstrated a gradual increase in Lassa fever‐related publications over the study period. In Scopus, scientific publication output increased rapidly between 2017 and 2025, but research output was comparatively low before 2017. The highest number of publications was over 140 in 2024 (Figure 2a). Similarly, WoS records showed a progressive increase in publication activity from 2001, although there was a decline in 2002. The research output increased progressively from 2003 to a peak of over 160 publications in 2024 (Figure 2b).

### 3.2. Most Contributing Countries and Their Collaborations

Authors′ affiliations were analyzed to identify the most prolific countries and institutions. Among the top 20 countries that contributed to Lassa fever research output within the study period, the United States and Nigeria occupied the first two positions in both databases (Table 1). In Scopus, the United States and Nigeria contributed 807 (45.4%) and 303 (17.1%) papers, whereas in WoS, the United States and Nigeria contributed 1243 (50.7%) and 324 (13.2%) papers, respectively. Among other African countries, Sierra Leone, Guinea, South Africa, Ghana, and Liberia contributed 107 (6.0%), 47 (2.6%), 38 (2.1%), 30 (1.7%), and 22 (1.2%) papers, respectively, in Scopus. In WoS, Sierra Leone, South Africa, Guinea, and Ghana contributed 120 (4.9%), 71 (2.9%), 57 (2.3%), and 36 (1.5%) papers, respectively.

**Table 1:** Top 20 countries with publications on Lassa fever as indexed in Scopus and WoS (1975–2025).

Rank	Scopus		WoS	
	Country	Publications, ***n*** (%) (***N* = 1776**)^a^	Country	Publications, *n*(%) (*N* = 2451)^a^
1^st^	United States	807 (45.4)	United States	1243 (50.7)
2^nd^	Nigeria	303 (17.1)	Nigeria	324 (13.2)
3^rd^	Germany	247 (13.9)	Germany	311 (12.7)
4^th^	United Kingdom	219 (12.3)	England	305 (12.4)
5^th^	China	109 (6.1)	France	159 (6.5)
6^th^	Sierra Leone	107 (6.0)	United Kingdom	139 (5.7)
7^th^	France	102 (5.7)	People′s Republic of China	133 (5.4)
8^th^	Canada	74 (4.2)	Sierra Leone	120 (4.9)
9^th^	Switzerland	72 (4.1)	Canada	111 (4.5)
10^th^	Japan	62 (3.5)	Switzerland	110 (4.5)
11^th^	Belgium	52 (2.9)	Japan	82 (3.3)
12^th^	Guinea	47 (2.6)	Belgium	77 (3.1)
13^th^	India	39 (2.2)	South Africa	71 (2.9)
14^th^	South Africa	38 (2.1)	Guinea	57 (2.3)
15^th^	Ghana	30 (1.7)	Argentina	52 (2.1)
16^th^	Netherlands	27 (1.5)	India	48 (2.0)
17^th^	Saudi Arabia	27 (1.5)	Netherlands	38 (1.6)
18^th^	Italy	25 (1.4)	Australia	37 (1.5)
19^th^	Australia	24 (1.4)	Ghana	36 (1.5)
20^th^	Liberia	22 (1.2)	Italy	35 (1.4)

^a^ *N* = total number of English‐language original research articles included in the bibliometric analysis after application of the eligibility criteria (Scopus = 1776; WoS = 2451); *n* = number of publications attributed to each country. Percentages were calculated using *N* as the denominator. Country counts were based on the full‐counting approach; therefore, publications with authors from multiple countries were counted once for each participating country. Consequently, totals may exceed the number of publications.

**Figure 3:**
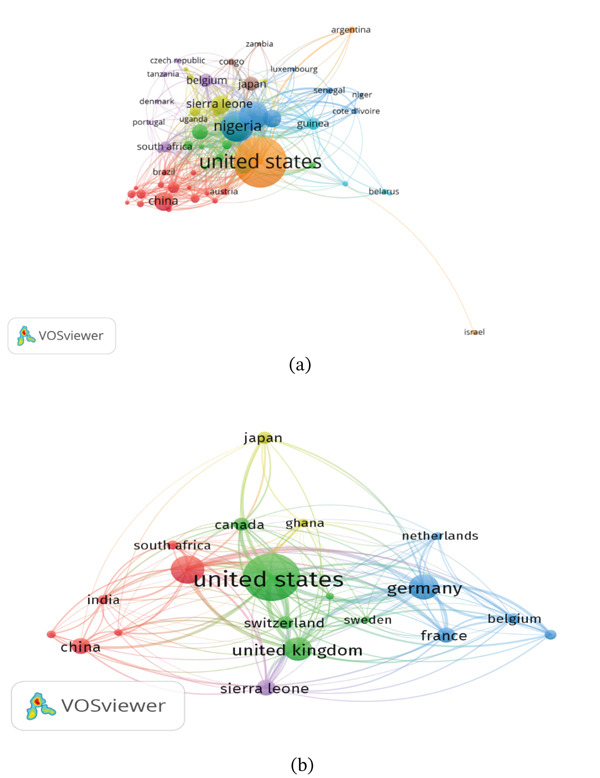
Network visualization of collaborations among countries in Lassa fever research. (a) Scopus and (b) WoS (1975–2025).

From both databases, the most crucial countries that showed collaborations with other countries in Lassa fever research were the United States and Nigeria in Scopus, and the United States, Germany, and the United Kingdom in WoS (Figure 3).

### 3.3. Prolific Institutions and Their Research Contributions

**Figure 4:**
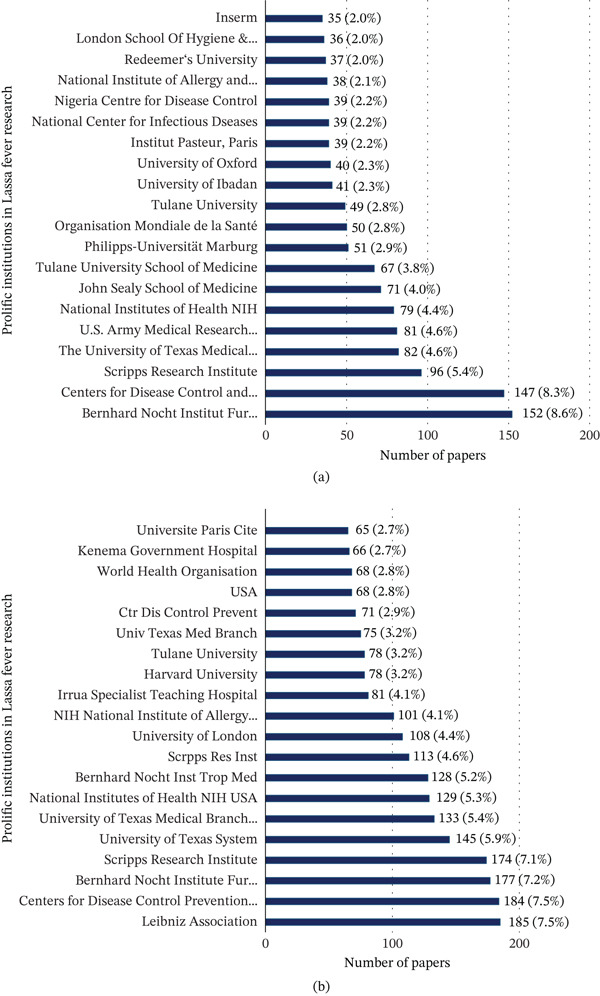
Most prolific institutions in Lassa fever research. (a) Scopus and (b) WoS (1975–2025).

The most influential institutions from Scopus were the Bernhard‐Nocht‐Institut für Tropenmedizin (152, 8.6%), the Centers for Disease Control and Prevention (147, 8.3%), and the Scripps Research Institute (96, 5.4%). From WoS, the Leibniz Association (185, 7.5%), the Centers for Disease Control and Prevention (184, 7.5%), and the Bernhard‐Nocht‐Institut für Tropenmedizin (177, 7.2%) topped the list (Figure 4).

### 3.4. Most Influential Funding Agencies and Research Fields

**Figure 5:**
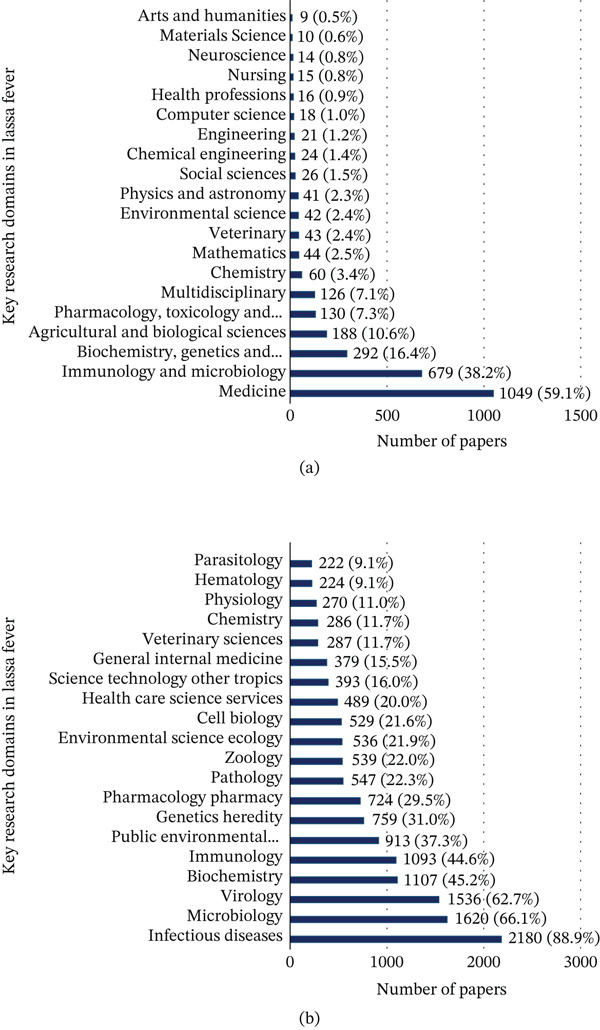
Most crucial research fields in Lassa fever research studies. (a) Scopus and (b) WoS (1975–2025).

The results show that from both databases, US agencies were the dominant funders of Lassa fever studies. These included the National Institute of Allergy and Infectious Diseases (318, 17.9%) and the National Institutes of Health (225, 12.7%), among other agencies in Scopus, whereas the US Department of Health and Human Services (647, 26.4%) and the National Institutes of Health (633, 25.8%) were among the agencies in WoS (Table 2). The most crucial research fields for Lassa fever research were categorized according to the Scopus and WoS fields. The top fields included “Medicine” (1049, 59.1%), “Immunology and Microbiology” (679, 38.2%), and “Biochemistry, Genetics and Molecular Biology” (292, 16.4%), among others, for Scopus documents. On the other hand, “Infectious Diseases” (2180, 88.9%), “Microbiology” (1620, 66.1%), and “Virology” (1536, 62.7%), among others, were the most crucial research fields within WoS (Figure 5).

**Table 2:** Top 20 funding agencies of Lassa fever research as indexed in Scopus and WoS (1975–2025).

Rank	Scopus		WoS	
	Funding agencies	Number of papers, *n*(%) (*N* = 1776)^a^	Funding agencies	Number of papers, *n*(%) (*N* = 2451)^a^
1^st^	National Institute of Allergy and Infectious Diseases	318 (17.9)	US Department of Health and Human Services	647 (26.4)
2^nd^	National Institutes of Health (United States)	225 (12.7)	National Institutes of Health	633 (25.8)
3^rd^	German Research Foundation (DFG)	76 (4.3)	National Institute of Allergy and Infectious Diseases	551 (22.5)
4^th^	National Natural Science Foundation of China	43 (2.4)	German Research Foundation (DFG)	116 (4.7)
5^th^	Wellcome Trust	40 (2.3)	Wellcome Trust	77 (3.1)
6^th^	European Union	37 (2.1)	UK Research and Innovation	75 (3.1)
7^th^	US Department of Health and Human Services	35 (2.0)	National Institute of General Medical Sciences	65 (2.7)
8^th^	Horizon 2020—Research and Innovation	32 (1.8)	European Union	62 (2.5)
9^th^	Coalition for Epidemic Preparedness Innovations	27 (1.5)	Defense Threat Reduction Agency	60 (2.4)
10^th^	National Key Research and Development Program of China	27 (1.5)	National Natural Science Foundation of China	51 (2.1)
11^th^	National Institute of General Medical Sciences	26 (1.5)	World Health Organization	50 (2.0)
12^th^	Japan Society for the Promotion of Science	25 (1.4)	Ministry of Education, Culture, Sports, Science, and Technology—Japan	47 (1.9)
13^th^	Centers for Disease Control and Prevention	23 (1.3)	US Public Health Service	46 (1.9)
14^th^	Medical Research Council	23 (1.3)	Medical Research Council	43 (1.8)
15^th^	7^th^ Framework Program for Research	22 (1.2)	Japan Society for the Promotion of Science	42 (1.7)
16^th^	Defense Threat Reduction Agency	21 (1.2)	National Cancer Institute	42 (1.7)
17^th^	US National Science Foundation	21 (1.2)	Swiss National Science Foundation	36 (1.5)
18^th^	Japan Agency for Medical Research and Development	19 (1.1)	National Institute of Neurological Disorders and Stroke	33 (1.3)
19^th^	Swiss National Science Foundation	19 (1.1)	Bill and Melinda Gates Foundation	31 (1.3)
20^th^	Bill and Melinda Gates Foundation	18 (1.0)	National Center for Research Resources	30 (1.2)

^a^ *N* = total number of English‐language original research articles included in the bibliometric analysis after application of the eligibility criteria (Scopus = 1776; WoS = 2451); *n* = number of publications attributed to each funding agency. Percentages were calculated using *N* as the denominator. As a single publication may acknowledge multiple funding agencies, each funding agency received one count per acknowledge publication; therefore, the cumulative funding counts may exceed the total number of publications.

### 3.5. Most Contributing Authors and Their Collaborations

A total of 8994 authors produced the analyzed publications from Scopus, whereas 9494 authors were identified in the WoS database. The top three productive authors of Lassa fever research were Günther S., Garry R.F., and Fichet‐Calvet E., each with 71, 60, and 50 papers and 4814, 1422, and 2288 total citations, respectively. In WoS, the top three productive authors of Lassa fever research were Günther S., De La Torre J.C., and Garry R.F., each with 114, 85, and 72 papers and 4864, 1472, and 2338 total citations, respectively (Table 3).

**Table 3:** Top 20 productive authors of Lassa fever infection research as indexed in Scopus and WoS (1975–2025).

Rank	Scopus			WoS		
	Authors	Total citations	Number of papers	Authors	Total citations	Number of papers
1^st^	Günther, S.	4814	71	Günther, S.	4864	114
2^nd^	Garry, R.F.	1422	60	De La Torre, J.C.	1472	85
3^rd^	Fichet‐Calvet, E.	2288	50	Garry R.F.	2338	72
4^th^	McCormick, J.B.	2296	42	Fichet‐Calvet, E.	2346	59
5^th^	Grant, D.S.	541	41	Lukashevich, I.S.	591	53
6^th^	Branco, L.M.	786	38	McCormick, J.B.	836	53
7^th^	Lukashevich, I.S.	1302	38	Grant, D.S.	1352	49
8^th^	Oestereich, L.	554	34	Barron, S.	260	52
9^th^	Schieffelin, J.S.	371	33	Kunz, S.	308	51
10^th^	Feldmann, H.	481	32	Campbell, K.P.	1454	49
11^th^	Goba, A.	712	32	Grant, D.S.	541	49
12^th^	Momoh, M.	376	30	Becker‐Ziaja, B.	1098	45
13^th^	Baize, S.	421	29	Ly, H.	651	45
14^th^	Kunz, S.	258	29	Cashman, K.A.	439	45
15^th^	Ogbaini‐Emovon, E.	139	29	Feldmann, H.	531	44
16^th^	Sabeti, P.C.	352	29	Branco, L.M.	836	43
17^th^	Odia, I.	216	28	Kuhn, Jens H.	2116	65
18^th^	Okogbenin, S.	221	28	Jahrling, P.B.	781	43
19^th^	Jahrling, P.B.	786	27	Mateo, M.	352	42
20^th^	Strecker, T.	222	27	Goba, A.	762	42

**Figure 6:**
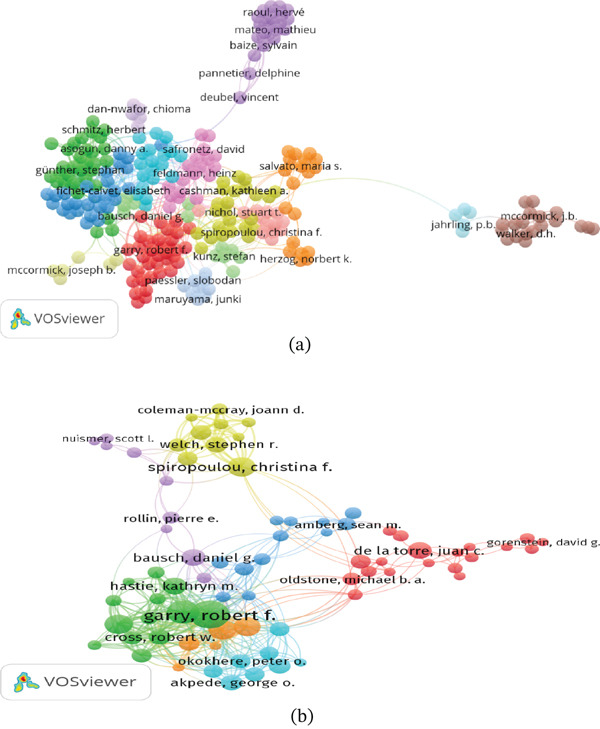
Co‐authorship analysis of the most influential authors in Lassa fever research. (a) Scopus and (b) WoS (1975–2025).

Analysis of the Scopus dataset revealed key collaborative research clusters involving Günther S., Garry R.F., and Fichet‐Calvet E., among other prominent researchers (Figure 6a). In the WoS database, significant collaborative networks in Lassa fever research were observed primarily between Garry R.F., Cross R.W., and De La Torre J.C., among other contributors (Figure 6b). These collaborations exemplified the interconnectedness and knowledge exchange that exist among the key researchers in the field.

### 3.6. Most Productive Journals

From Scopus, the analyzed documents were published by 1764 journals, whereas those from WoS were produced by 2451 journals. The 20 most productive journals from both databases are listed in Table 4, where the *Journal of Virology* topped the lists of both databases.

**Table 4:** Top 20 journals of Lassa fever from Scopus and WoS (1975–2025).

Rank	Scopus			WoS		
	Journal name	No. of papers (*N* = 1776)	Impact factor (2025)	Journal name	No. of papers (*N* = 2451)	Impact factor (2025)
1^st^	*Journal of Virology*	135	4.1	*Journal of Virology*	170	4.1
2^nd^	*PLOS Neglected Tropical Diseases*	58	3.4	*Viruses*	89	3.8
3^rd^	*Viruses*	49	3.8	*PLOS Neglected Tropical Diseases*	70	3.4
4^th^	*Emerging Infectious Diseases*	47	6.6	*Antiviral Research*	60	4.3
5^th^	*Antiviral Research*	37	4.3	*Emerging Infectious Diseases*	60	6.6
6^th^	*Virology*	33	2.4	*Virology*	56	2.4
7^th^	*Journal of Infectious Diseases*	32	4.1	*American Journal of Tropical Medicine and Hygiene*	54	1.6
8^th^	*American Journal of Tropical Medicine and Hygiene*	31	1.6	*Journal of Infectious Diseases*	53	4.1
9^th^	*Plos One*	31	2.8	*American Journal of Pathology*	53	4.7
10^th^	*Plos Pathogens*	30	4.9	*PLOS Pathogens*	42	4.9
11^th^	*Bulletin of the World Health Organization*	28	8.4	*PLOS One*	38	2.8
12^th^	*Scientific Reports*	28	4.9	*Scientific Reports*	37	4.9
13^th^	*Transactions of the Royal Society of Tropical Medicine and Hygiene*	28	1.6	*Bulletin of the World Health Organization*	35	8.4
14^th^	*Lancet*	21	109.0	*Transactions of the Royal Society of Tropical Medicine And Hygiene*	34	1.6
15^th^	*Proceedings of the National Academy of Sciences of the United States of America*	20	9.1	*Journal of General Medicine*	33	3.8
16^th^	*Nature Communications*	19	18.1	*Vaccine*	33	3.5
17^th^	*Vaccine*	19	3.5	*Nature Communications*	31	18.1
18^th^	*Virology Journal*	19	4.0	*Current Opinion in Infectious Diseases*	28	3.6
19^th^	*Journal of General Virology*	18	3.8	*BMJ British Medical Journal*	22	55.1
20^th^	*Journal of Clinical Microbiology*	16	5.9	*Proceedings of the National Academy of Sciences of the United States of America*	20	9.1

### 3.7. Keyword Analysis

**Figure 7:**
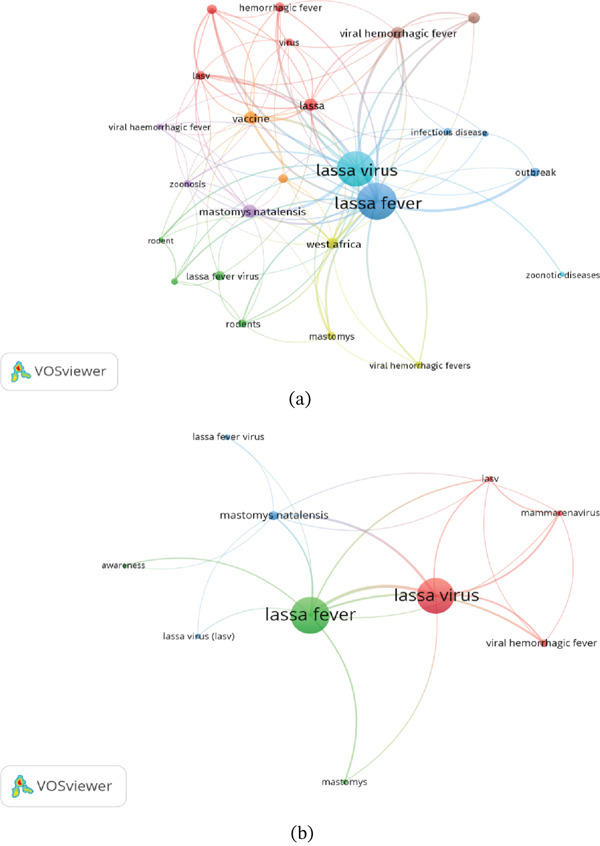
Visualization of keywords as indexed in Lassa fever research. (a) Scopus and (b) WoS (1975–2025).

On analysis, Scopus documents had 12,367 Keywords Plus and 2668 author keywords, whereas WoS documents had 12,867 Keywords Plus and 3168 author keywords. Keyword distribution was analyzed to detect directions and topics in Lassa fever research and to understand discipline development. The most frequent author keywords were “Lassa fever” and “Lassa virus,” with over 200 occurrences in both databases (Figure 7a,b).

## 4. Discussion

This bibliometric analysis of Lassa fever presents updated insights into its dynamic evolution over the years in terms of trends and scientific publication output. The initial search revealed that 87.0% and 89.6% of the English publications were retrieved from the Scopus and WoS databases, respectively. English is the most widely used official language for international scientific communication, a finding that corroborates earlier studies [1–3]. Another reason for the high output in English could be that during disease outbreaks, scientific findings and communications occur predominantly in English, facilitating rapid international dissemination of scientific evidence [5, 6].

Trends in Lassa fever publication output show a rapid increase from 2017 with a peak in 2024 (in Scopus) and from 2003 with a peak in 2024 (in WoS). This coincides with recurrent Lassa fever outbreaks in West Africa, particularly Nigeria, which have stimulated increased research activities and publication output [10, 11, 16, 17, 19–25]. Notably, the sharp rise in publication output from 2017 onwards closely followed the major Lassa fever outbreaks reported in Nigeria during 2018, 2019, and 2020, which were characterized by substantial increases in confirmed cases, enhanced surveillance, and strengthened national response activities. The 2018 outbreak represented one of the largest recorded Lassa fever epidemics in Nigeria and stimulated intensified research into the epidemiology, diagnosis, clinical management, molecular characterization, and prevention of the disease. Continued outbreaks in 2019 and 2020 sustained research interest and investment, contributing to the sustained increase in publication output observed through 2024. These findings suggest that recurring outbreaks served as important drivers of scientific activity and international collaboration in Lassa fever research [17, 22–24]. Similar concerns have been reported following imported Lassa fever cases outside endemic regions, which have reinforced the need for international preparedness and surveillance [10, 12].

Among the top countries contributing to Lassa fever research, the United States and Nigeria dominated the lists in both databases. Sierra Leone, Guinea, South Africa, and Ghana appeared among the top 20 productive countries. The results imply that the United States has maintained its leading role in not only Lassa fever research but also in other research fields. Nigeria′s emergence as a leading contributor reflects vital local engagement, which is essential for designing interventions tailored to regional contexts. Nigeria′s increased scientific contribution is consistent with its position as the country bearing the highest burden of Lassa fever and experiencing frequent outbreaks, making local research indispensable for improving surveillance, diagnosis, and disease control [8, 10, 11, 16, 24, 25].

Furthermore, the most influential affiliations included the Bernhard‐Nocht‐Institut für Tropenmedizin, the Centers for Disease Control and Prevention, the Scripps Research Institute, the Leibniz Association, and the Centers for Disease Control and Prevention (United States), among others. Despite the endemicity of Lassa fever in West Africa, only a few African institutions were represented among the top 20 most productive institutions. In Scopus, these included the Nigeria Centre for Disease Control, Redeemer′s University, and the University of Ibadan, whereas the WoS identified Irrua Specialist Teaching Hospital and Kenema Government Hospital among the top 20 institutions. Given that Lassa fever disproportionately affects Nigeria and neighboring countries, greater representation of African institutions in global research is expected and should be encouraged through increased investment in local scientific capacity [8, 10, 11].

Network analysis showed that the Bernhard‐Nocht‐Institut für Tropenmedizin, the CDC, the Scripps Research Institute, and the Department of Microbiology and Immunology exhibited the highest degree of collaboration within Lassa fever research. Additionally, the analysis showed that the United States takes the leading role in terms of collaboration. International collaboration has been instrumental in advancing genomic surveillance, molecular epidemiology, and therapeutic development for Lassa fever, particularly through partnerships between institutions in endemic countries and leading research centers in Europe and North America [12, 15, 19–21]. A few African countries also exhibited a significant degree of cooperation. However, there is a need for African countries to develop and improve their research facilities to enable them to carry out independent research.

Regarding funding for Lassa research, US and Western agencies predominantly topped the list, with no African agency among the top 20. These funding bodies do more than provide capital; they act as expertise hubs that foster interdisciplinary collaboration, facilitate the translation of research into clinical solutions, and raise global political awareness. This implies a need for more funding from African governments and institutions. Increased domestic investment would ensure better healthcare infrastructure and outbreak preparedness in order to benefit the most affected local communities. Increased domestic investment would strengthen laboratory capacity, genomic surveillance, clinical research, and outbreak preparedness within endemic countries, thereby reducing dependence on external funding agencies [12, 16, 17]. The primary Lassa fever research fields were “Medicine,” “Immunology and Microbiology,” “Infectious Diseases,” and “Microbiology,” among others. This multidisciplinary distribution reflects the complex nature of Lassa fever, encompassing epidemiology, virology, immunology, molecular biology, clinical medicine, therapeutics, and public health [14, 15, 20, 21].

The study results identified the most contributing authors of Lassa fever research, which included Günther S., Garry R.F., Fichet‐Calvet E., and De La Torre J.C., among others. Furthermore, network analysis revealed authors with the highest collaborations. These prolific authors underscore the necessity of a multidisciplinary approach and provide a roadmap for future researchers to identify potential partners. Many of these authors have contributed substantially to understanding Lassa virus biology, molecular evolution, pathogenesis, diagnostics, and clinical management, explaining their prominent positions within collaborative research networks [12, 14, 15, 20]. This information would be helpful to future researchers in identifying crucial possible researchers for partnership or even consultation.

Additionally, the analysis noted that the top journals for Lassa fever research are foreign journals, including the *Journal of Virology*, *PLOS Neglected Tropical Diseases*, and *Viruses*, among others. This implies that most of the valuable findings and recommendations on Lassa fever research are published in foreign journals. These journals have historically published many of the landmark studies on Lassa fever, including investigations of viral ecology, molecular evolution, pathogenesis, clinical management, and therapeutic development [7, 8, 12, 14, 15, 21]. The recommendations in these papers may not be freely or easily accessible to African researchers. Therefore, there is a need for African countries to improve and strengthen local research databases and journals.

Keyword analysis revealed the most common keywords which included “Lassa fever” and “Lassa virus,” among others. The prominence of these keywords reflects sustained scientific interest in the epidemiology, virology, and clinical management of Lassa fever over several decades [8, 10, 11, 14, 15]. The dominance of these keywords reflects the efforts put into formulating and testing new effective strategies targeted towards the prevention, management, and treatment of Lassa fever. Furthermore, the presence of other topics such as “mammarenavirus,” “*mastomysnatalensis*,” and “viral hemorrhagic fever” indicates that Lassa fever is a zoonotic disease, which can be transmitted by rats to the human population. The frequent occurrence of keywords such as “mastomysnatalensis” and “mammarenavirus” further highlights the continued importance of reservoir ecology, zoonotic transmission, viral evolution, and molecular epidemiology in shaping contemporary Lassa fever research [13, 14, 20].

Overall, the findings of this study demonstrate that Lassa fever research has expanded considerably over the past five decades but remains characterized by marked geographical disparities in research productivity, funding, and institutional participation. International collaboration has accelerated scientific advances in understanding the virus and improving disease management. However, greater investment in research infrastructure, funding mechanisms, and collaborative networks within endemic African countries is essential to ensure sustainable scientific development and strengthen regional capacity to prevent and control future Lassa fever outbreaks.

## 5. Limitations

Unlike previous similar bibliometric studies, this one concurrently analyzed and compared two databases (WoS and Scopus). This bibliometric analysis on Lassa fever research has several limitations. First, the findings were dependent on the search strategy employed. Although the search string was carefully developed using relevant keywords and Boolean operators to maximize sensitivity and specificity, it may not have captured all eligible publications because some studies may have used alternative terminology, abbreviations, spelling variations, or indexing terms that were not included in the search query. Consequently, some relevant studies may have been inadvertently omitted. The analysis was also limited to English‐language research articles indexed in Scopus and WoS. Although this approach ensured consistency in document type and facilitated the analysis of peer‐reviewed primary research, it may have excluded relevant studies published in French and other languages, particularly from Lassa fever‐endemic countries in Francophone West Africa. Consequently, the findings may be subject to language (Anglophone) bias and may not fully capture the regional diversity of Lassa fever research. Second, only original articles were included, whereas reviews, editorials, conference papers, letters, notes, and other document types were excluded to ensure a homogeneous dataset for bibliometric analysis and to minimize differences in publication and citation patterns across document types. Finally, despite the use of a comprehensive search strategy, the exclusion of other databases, such as PubMed, Embase, Dimensions, AGORA, ScienceDirect, African Journals Online (AJOL), and Google Scholar, together with the inherent limitations of the search strategy, may have resulted in the omission of some relevant publications not indexed in the selected databases. Therefore, the findings should be interpreted within the scope of the selected databases and inclusion criteria.

## 6. Conclusions

The current bibliometric analysis summarized Lassa fever research output (1975–2025). It therefore aids in highlighting sustained growth in scientific output, expanding international collaboration, and increasing multidisciplinary engagement. The study also identified the leading roles played by various top productive countries, authors, institutions, and journals. However, African research output is disproportionately low in relation to other countries. There is a critical need for increased investment in Lassa fever research by African governments and regional funding agencies. Strengthening local laboratory infrastructure, promoting regional and international collaborations, supporting open‐access publication, and expanding genomic surveillance programs could be essential for improving outbreak preparedness and advancing vaccine and therapeutic development. Policymakers should also prioritize capacity‐building initiatives aimed at enhancing indigenous research leadership in endemic regions.

## Author Contributions

A.N.O. helped in the conceptualization, design, and supervision of the study. I.F.C. and A.N.O. contributed to the critical appraisal of study eligibility and methodological rigor. C.M.O. helped in data curation. I.F.C. helped in investigations, software visualization, formal analyses, writing, and original manuscript draft preparation. S.P.Y.D., C.M.O., K.D.O., and R.A.A. provided senior intellectual input and contributed to manuscript review. S.P.Y.D. contributed to critical revisions, assisted with final manuscript edits, and provided substantive intellectual feedback.

## Funding

No funding was received for this manuscript.

## Disclosure

All authors reviewed and approved the final version of the manuscript.

## Ethics Statement

Not applicable. This study was based exclusively on the review of previously published literature and did not involve human participants or animal experimentation.

## Conflicts of Interest

The authors declare no conflicts of interest.

## Data Availability

The data supporting the findings of this study are included within this article. The bibliographic data analyzed during this study were retrieved from the Scopus and Web of Science Core Collection databases using the search strategy described in the Materials and Methods section and are available from these databases.
